# A Rare Cause of the Cough: Primary Small Cell Carcinoma of Esophagus—Case Report

**DOI:** 10.1155/2012/870783

**Published:** 2012-02-22

**Authors:** Erdal Yekeler, Timur Koca, Semra Vural

**Affiliations:** ^1^Department of Thoracic Surgery, Atatürk Chest Diseases and Thoracic Surgery, Training and Research Hospital, 25070 Ankara, Turkey; ^2^Department of Radiation Oncology, Region Training and Research Hospital, 25070 Erzurum, Turkey; ^3^Department of Pathology, Esenyurt State Hospital, 25070 Istanbul, Turkey

## Abstract

Primary small cell carcinoma of the esophagus is a relatively rare malignancy. It is highly progressive and poorly prognostic in untreated conditions. In the western populations, the rate of primary small cell carcinoma in all esophageal cancer types is between 0.05% and 2.4%, while it is endemically increasing up to 7.6% in the eastern populations. Most of the cases are in extensive stage at the time of diagnosis. Surgery is the treatment of choice in limited stages, but treatment must be multimodal in primary small cell carcinoma of the esophagus. A 47-year-old woman was referred to our clinic with gradually increasing severe dry cough and slight difficulty in swallowing for 20 days. Chest X-ray graphy was normal, and computed tomography of the chest showed multiple mediastinal lymph nodes and hepatic metastases. Her endoscopic examination revealed an endoluminal vegetative mass between 20 cm and 23 cm of her esophagus. The case was reported as small cell carcinoma of the esophagus on histopathological examination. The case was assumed inoperable, and chemotherapy and radiotherapy were planned. We presented a rare cause of the cough and primary esophageal small cell carcinoma in this paper.

## 1. Introduction

Small cell carcinomas (SCCs) are more often described in lungs, but rarely laryngeal, pancreatic, stomach, prostatic, uterine, sweet glands, and esophageal locations are reported [1, 2]. Esophageal and extrapulmonary small cell Carcinoma (EPSCC) was described first by McKeown in 1952 [3]. Primary small cell carcinoma of the esophagus (PSCCE) is a rare, rapidly progressive, and highly metastatic disease with poor prognosis. The incidence of PSCCE between all esophageal malignancies is from 0.05 to 2.4% in western populations, and this rate rises up to 7.6% in Chinese and Japanese literature [1, 4, 5]. As seen in our case, the cases with tracheal invasion due to rapid progression of PSCCE, without the presence of dysphagia in the foreground, admit to the hospital with the complaint of cough. From this aspect, we presented a case of extrapulmonary intrathoracic SCC, because it was both a rare etiology of severe dry cough and an indicator of rapid progression of PSCCE.

## 2. Case Report

**Figure 1:**
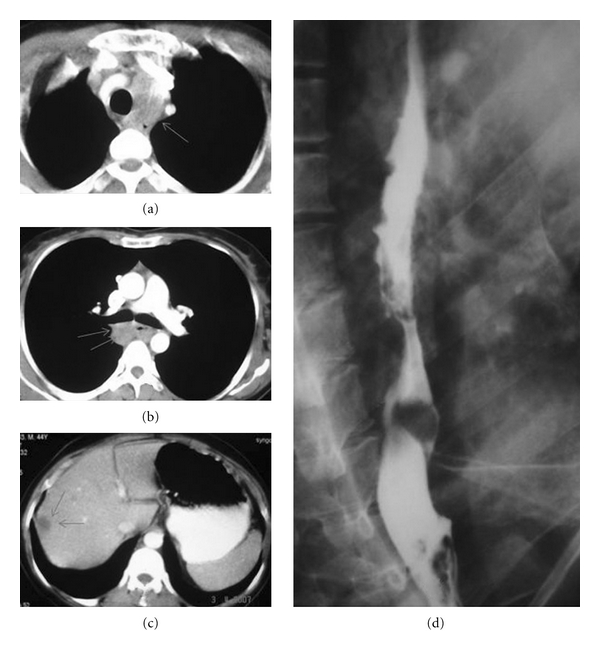
(a, b, c) Thorax CT revealed the multiple mediastinal lymph nodes up to 2-3 cm and hepatic metastasis (arrows). (d) The barium-contrasted esophageal graphy is showing mucosal irregularity.

**Figure 2:**
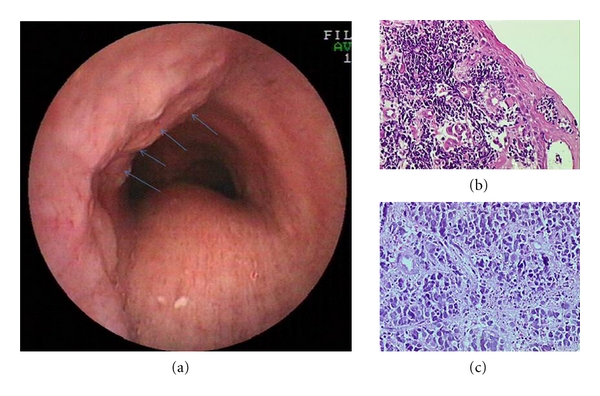
(a) Bronchoscopic exploration showed submucosal tumoral infiltration at the left anterolateral wall of trachea (arrows). (b, c) Biopsy materials are shown in the histopathologic examination.

A 47-year-old woman was referred to our clinic with gradually exacerbating dry cough and slight dysphagia for twenty days. There was no abnormality on the chest X-ray graphy. Thoracic computed tomography (CT) (Figures 1(a), 1(b), and 1(c)) revealed a mass and mediastinal multiple lymph nodes up to 2-3 cm and also hepatic metastases. Bronchoscopic exploration (Figure 2(a)) carried out for severe dry cough and to evaluate subcarinal mediastinal lymph node showed submucosal tumoral infiltration at the left anterolateral wall of the distal trachea. Esophageal endoscopic evaluation revealed an endoluminal vegetative mass between 20 and 23 centimeters of her esophagus. Barium-contrasted esophageal graphy (Figure 1(d)) showed mucosal irregularity and thickness in a long esophageal segment. Biopsy was obtained and pathological specimen reported as small cell carcinoma of esophagus. In the histopathologic examination (Figures 2(b) and 2(c)) of biopsy materials belonging to esophagus taken endoscopically from the patient, accumulations have formed in lamina propria without indicating remarkable glandular or squamous organization, and it was observed that there was neoplastic formation leading to small rounds in squamous epitelium sporadically. The cells, forming neoplastic formation in which intensive squeezed artefacts and mitotic figures were observed, were round and ovalshaped having this granular chromatin and had narrow cytoplasm, the boarding of which is not chosen well, and its nuclei do not appear as one on the top of the other. In immunohistochemical examination, these tumoral cells indicated chromogranin, synaptophysin, NSE, and CD-56 with a positive immunereactivity. Immuno-reactivity together with Pan-CK and LCA was not observed. The case in this shape condition was reported as PSCCE.

Chemotherapy and radiotherapy were planned in this case that was considered inoperable. Patient received concurrent chemotherapy and radiation therapy using a total dose of 50 Gry in 25 fractions, five fractions per week. The chemotherapy consisted of 75 mg/m^2^ cisplatinum given intravenously on the first day and 1 g/m^2^ 5-FU given by continuous infusion for the first 4 days of weeks 1, 5, 8, and 11. Patient was initiated to be administered radiotherapy and antitussive therapy, which led to the regression of the complaints. At the end of 6 months, a brain metastasis developed, and the patient was lost.

## 3. Discussion

SCC which constitutes 15–20% of all bronchial carcinomas mostly arises from lungs. EPSCCs are identified for other organs except esophagus. PSCCE is a rare tumour characterized by early dissemination and poor prognosis if untreated [1, 2, 5, 6].

East side of Turkey is an endemic region for esophageal cancers. For instance, its incidence has been reported as 3/100,000 in Europe and USA, while it is 165–200/100,000 in Eastern Turkey, Northern Iran, and China [7, 8]. Between October 2004 and January 2010, 294 patients with esophageal carcinoma were admitted and treated in our clinic with the therapies including esophageal resection, stent application, and conservative therapy in the patients treated with trachea-bronchial or esophagopleural fistula and chemoradiotherapy in the patients agreed to be inoperable. In the retrospective analysis, small cell carcinoma was found in only two cases (0.68%).

Endoscopic and radiological findings of PSCCE resemble squamous or adenocarcinoma of the esophagus. But progressive dysphagia, poor prognosis, rapid weight loss, and distant metastasis are against our interests in early period. Definitive diagnosis of PSCCE is diagnosed by cytological examination with esophageal abrasive balloon and endoscopic punch biopsy. This tumour is mostly reported in men with a male-to-female ratio reported as 2 : 1. It has often been reported between the fourth and the seventh decades. Major symptoms are progressive dysphagia, retrosternal pain, and rapid weight loss. In some cases, hoarseness and upper gastrointestinal tract bleeding have been reported as the primary symptoms. As seen in our case, even rarely, severe cough is the primary and leading symptom. Lesions are usually confined to middle and lower esophagi. Hematogeneous metastases of PSCCE are mainly extended to liver, lung, and bones [1, 2, 4, 5].

There are two viewpoints on the histological origin of PSCCE. The first is that PSCCE originates from neuroendocrine cells of the submucosal gland or stratum basal, that is, the major precursor uptake and decarboxylation cells, as histologically confirmed. The second is that PSCCE originates from multipotential stem cells of the endoderm. Most of these cells may be differentiated into squamous cell carcinoma, and some are differentiated into adenocarcinoma or small cell carcinoma. This is due to the diversity of morphological, immune-histological, and electron microscopic features of PSCCE, in addition to the coexistence of PSCCE with squamous cell carcinoma and/or adenocarcinoma [5].

The standard of treatment for PSCCE has not been established yet due to the paucity of cases. Treatments such as operation alone [6], local radiotherapy [9], chemotherapy alone [10], or operation with adjuvant therapy [11] have been reported. In the limited disease, after surgical resection, short-term results of chemotherapy and radiotherapy are good, although long-term results are still poor. In a series of 29 patients with limited disease treated with only surgery, average survival was 8 months [12]. Also in a series of 20 patients with limited disease patients treated only with radiotherapy, average survival was 5 months [13]. After the basis of biological behavior, chemosensitivity, radiosensitivity, and some satisfaction in the treatment of small cell lung carcinomas, systemic chemotherapeutic agents PSCCE came to the fore. In early detected cases, surgical resection combined with radiotherapy and chemotherapy is the best way to treat PSCCE. In advanced stages, multiagent chemotherapy is the treatment of choice, and radiotherapy can be used for palliation.

PSCCE is an extremely rare, rapidly progressive, and highly malignant characterised esophageal pathology and prone to early metastasis. In these cases, treatment must be quickly decided and started as soon as possible. The treatment is multimodal. Surgery is the standard treatment in limited stages. In advanced stages, radiotherapy with multiagent chemotherapy is a treatment choice. Despite all treatment principals, prognosis is still poor in these cases. As in our case, it is possible to detect newly and less symptomatic patients in advanced stages. In these cases we believe that multiagent chemotherapy and radiotherapy are correct treatment options.

Approximately 5% of all the small cell carcinomas are extrapulmonary. Extrapulmonary small cell carcinoma (EPSCC) is called as limited disease (LD) and extensive disease (ED) as in pulmonary SCC. LD was defined as a localized tumour with or without regional lymph node involvement. The cases with distant organ or lymph node invasion referred to ED. Treatment protocols in EPSCC are similar to those in lungs and can be treated with cisplatinum-based regimens for chemotherapy. Surgery is of benefit in LD. Multimodal therapy including chemotherapy and radiotherapy should be preferred in EPSCC even if the diagnosis was established in the early period and surgery was performed. In 34 EPSCC cases studied by KO Kim et al., 23 of the cases had LD and 11 had ED, and 6 (17,6%) of these were reported as esophageal origin 6 (17,6%) and as thymus origin 6 (17,6%). Ten cases with LD underwent surgery. Overall survival was found as 19,8 months in LD and 7 months in ED. Overall survival was estimated as 14 months for all the cases. Multimodal therapy principles were applied depending on the patient's suitability both in LD and ED cases. The most commonly used chemotherapy regimen was the combination of etoposide and platinum compounds (cisplatin or carboplatin) [14].

Extrapulmonary-intrathoracic SCC (esophageal, thymus, etc.) and pulmonary SCC are rapidly progressive malignancies [14]. As observed in our case which was ED, it can be metastatic while newly symptomatic. In a healthy individual, persistent cough should always be taken into account. Similarly to pulmonary small cell carcinoma, esophageal small cell carcinoma remains to be a challenge for medical therapy.
